# Optimizing drugs to reach treatment targets for children and adolescents living with HIV

**DOI:** 10.7448/IAS.18.7.20270

**Published:** 2015-12-02

**Authors:** Martina Penazzato, Janice Lee, Edmund Capparelli, Shaffiq Essajee, Nathan Ford, Atieno Ojoo, Fernando Pascual, Nandita Sugandhi, Marc Lallemant

**Affiliations:** 1 HIV Department, World Health Organization, Geneva, Switzerland; 2 Drugs for Neglected Diseases Initiative, Geneva, Switzerland; 3 Clinical pharmacology, University of California, San Diego, CA, USA; 4 UNICEF, Copenhagen, Denmark; 5 Medicine Patent Pool, Geneva, Switzerland; 6 Clinton Health Access Initiative, Boston, MA, USA

**Keywords:** antiretrovirals, treatment, children, ART, optimization, formulations, HIV

## Abstract

**Introduction:**

As the global community makes progress towards the 90-90-90 targets by 2020, a key challenge is ensuring that antiretroviral drugs for children and adolescents are suitable to the context of resource-limited settings. Drug optimization aims to support the expanded use of more simplified, less toxic drug regimens with high barriers to drug resistance that require minimal clinical monitoring while maintaining therapeutic efficacy. This manuscript summarizes the progress made and outlines further critical steps required to ensure that the right drugs are available to start children and adolescents on treatment and to keep them virologically suppressed.

**Discussion:**

Building upon previous work in drug optimization, several important steps were taken in 2014 to ensure alignment between WHO dosing recommendations and the requirements of regulatory bodies, to accelerate drug development, to reduce intellectual property barriers to generic production of combined formulations and rationalize drug selection in countries. The priority for the future is to improve access to antiretroviral therapy (ART) at the two ends of the paediatric age spectrum – infants and adolescents – where the treatment gap is greatest, and optimize drug sequencing with better use of available medicines for second- and third-line ART. Future efforts in this area will require continuous collaboration and coordination, and the promotion of innovative approaches to accelerate access to new drugs and formulations.

**Conclusions:**

While significant progress has been made, additional efforts are needed to ensure that treatment targets are reached by 2020.

## Introduction

The world has recently achieved the global treatment target of reaching 15 million people in antiretroviral therapy (ART) by 2015 and countries are now moving ahead towards reaching the new 90-90-90 targets [1]. However, improvements in access to treatment have been greater for adults than for children. There are many reasons for this, but a fundamental challenge is the enduring complexity of treatment regimens, particularly for young children. For adolescents, high rates of non-adherence to treatment and loss to care point to the need to prioritize simpler and more tolerable regimens.

Paediatric HIV is largely a disease affecting children in resource-limited settings [2], and as a result children have not benefited from the same level of engagement in drug development as adults. Research and development (R&D) of new drugs for children has historically been complex and time consuming, with drug approvals for children lagging years behind the approval for adults – up to nine years in the case of tenofovir (TDF), for example [3]. Without dedicated efforts, the situation can be expected to get worse. The reduction in new infections resulting from the rapid expansion of more effective interventions to prevent mother-to-child transmission with 220,000 new infections occurring in 2014 [4] has reduced the paediatric market and further dis-incentivized manufacturers to invest in paediatric antiretroviral (ARV) R&D.

Unfortunately, current estimates suggest that despite increasingly successful implementation of PMTCT interventions over 1 million children will still be in need of treatment in 2020 [5]. This paper summarizes some of the major milestones and advances in treatment optimization for children and adolescents, and aims to outline the critical steps needed to ensure that the right drugs are available so that children and adolescents can start on treatment promptly and maintain virological suppression.

## Discussion

### The path towards drug optimization

Early efforts to optimize treatment for patients in resource-limited settings focused on the development of nevirapine (NVP)-based fixed-dose combinations (FDCs) in adults. The development of these FDCs, with no pre-existing FDA or EMEA originator products, required an innovative regulatory framework to review these dossiers [6].

In the absence of suitable alternatives, adult FDCs were initially used to treat children, often with limited evidence regarding appropriate dosing. Building upon the early efforts by MSF and others in simplifying dosing and using weight-bands [7], WHO convened a technical meeting in 2005 to harmonize global paediatric dosing recommendations. This meeting led to the development of the WHO generic tool for assessing formulation dosing strategies using existing ARVs. Exposure targets were based primarily on the labelled dose and incorporating additional knowledge of maturational changes in drug metabolism [8]. This weight-band dosing approach for individual ARVs and FDCs was included in the WHO 2006 and 2010 guidelines for the treatment of infants and children with HIV within a public health approach [9].

The next revision of WHO treatment guidelines, released in 2013 [10], represented another milestone for drug optimization, rationalizing the number of treatment options to simplify procurement and prescribing, recommending more potent drugs for young children, and harmonizing recommendations with adult regimens down to 10 years of age. These guidelines recognized that different approaches to treat younger children were still required because of differences in the natural history of HIV infection, and differing pharmacokinetics (PK), efficacy and toxicity profiles of ARVs depending on age. However, while the WHO 2013 Guidelines have been widely adopted for adults, the implementation of paediatric drug recommendations has been slow and challenging in most countries due to the lack of age-appropriate formulations for key drugs such as LPV/r (which has an unpalatably high alcohol content and requires cold chain storage until dispensing) and TDF (which remains unaffordable for most settings and is currently only available in powder and multiple single-compound dosage forms from the originators).

Consequently, the most common ARV regimen used for children remains AZT/3TC/NVP [11]. Analysis of procurement patterns suggests that the main driver in product selection is the availability of dual or triple dispersible FDC rather than their cost or efficacy [12].

### Accelerating actions to optimize treatment for children

**Figure 1:**
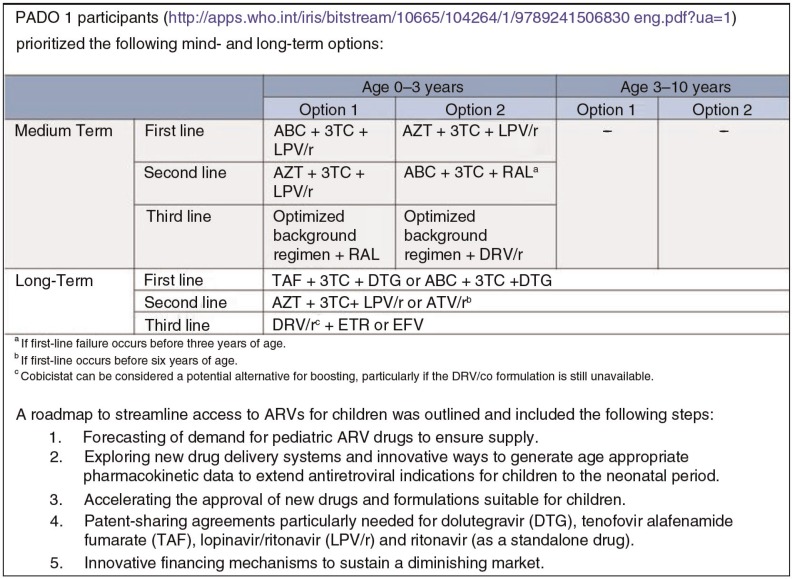
Medium- and long-term priorities for drug sequencing in children [13].

Building upon previous work in drug optimization (PADO 1; Figure 1), important further steps were taken in 2014 to ensure alignment between WHO dosing recommendations and the requirements of regulatory bodies, as well as to reduce intellectual property barriers and accelerate drug development and rationalize drug selection in countries [14].

#### Optimizing the use of ARVs for children

Experts from the WHO Paediatric ARV Working Group (PAWG) have provided evidence-based recommendations to guide age-appropriate dosing. The WHO generic tool was revised to reflect the non-linear relationship between weight and drug bioavailability due to organ maturation in young infants [15]. Population-based PK models incorporating the maturational changes in absorption, distribution, metabolism and clearance have been used together with simulations to assess ARV exposure (trough concentrations and AUCs) obtained when following WHO weight-band dosing recommendations. Concerns about potential under-dosing required a revision of the WHO weight-band dosing for DRV and DRV/r (Table 1). Also, a collaborative PK modelling was undertaken to establish the appropriate dosing for a new FDC including ABC/3TC/EFV. This additional modelling was undertaken in light of the high variability of EFV PK and the concerns raised about potential under-dosing observed in African trials [16] where WHO weight-band dosing was used. This has now resulted in more robust dosing recommendations that will guide the development of this FDC.

**Table 1:** PAWG-recommended weight-band dosing for DRV single compound and DRVr co-formulation to be used twice daily as part of second- or third-line regimens*

		Number of tablets/mL by weight-band morning and evening					
		
Drugs	Strength of paediatric tab (mg)	3–5.9 kg	6–9.9 kg	10–13.9 kg	14–19.9 kg	20–24.9 kg	25–29.9 kg
DRV^a^	100 mg/mL	NR	NR	2.5 (250 mg)	3.5 (350 mg)	–	–
DRV^a^	75 mg tablets	NR	NR	3 (225 mg)	5 (375 mg)	5 (375 mg)	5 (375 mg)
DRV/r	120/20*	NR	NR	2 (240 mg)	3 (360 mg)	3 (360 mg)	4 (480 mg)

a DRV single-strength dosing must be used with the appropriate dosing of RTV.

#### Developing priority medicines for children

The need to overcome intellectual property barriers that prevent individual drugs developed by different drug companies to be assembled in a potential FDC and to navigate the existing regulatory pathway for formulations which lack originator equivalent led to the establishment of the Paediatric HIV Treatment Initiative (PHTI) [17]. This initiative aims to accelerate the development of WHO-recommended paediatric ARV formulations by coordinating drug development and promoting increased access by engaging with industry to ensure sharing of intellectual property rights and know-how, and facilitate formulation development. Two drug development projects were launched: ABC/3TC/EFV and DRV/r.

#### Optimizing selection and procurement of existing paediatric products

In recent years, a number of improved ARV formulations have become available, such as dispersible FDCs in place of the traditional liquid formulations. These products have greatly simplified paediatric HIV care in low-income settings; however, the proliferation of these newer options, in addition to the availability of older sub-optimal products has resulted in a multiplicity of formulations across regimens and weight-bands. The fragmentation of demand across too many similar products has in some cases led to stock-outs as there was not enough demand for manufacturers to produce sufficient quantities of low-volume products.

For this reason, the Interagency Task Team on prevention and treatment of HIV infection in pregnant women, mothers and their children (IATT – convened by WHO and UNICEF in collaboration with multiple implementing partners) provided formulary guidance to programmes on selection of optimal paediatric ARVs. The formulary was first developed in 2011, revised in 2013 [18] using a more robust set of criteria (Table 2) and updated again in December 2014. Currently, the optimal formulary contains nine paediatric ARVs, plus a “limited use list” containing products that may be needed under special circumstances, or products that are transitioning in or out of the optimal formulary [19].

**Table 2:** Criteria for evaluation of paediatric ARV products included in IATT optimal formular [18]

Criterion	Definition	
Meets WHO requirements	•	Included in the latest WHO guidelines for paediatric treatment
Allows for widest range of dosing options	•	Allows for flexible dosing across multiple weight-bands and ages
Approved by SRA/WHO PQ	•	Availability of at least one SRA-approved product
User friendly	•	Easy for healthcare worker to prescribe
	•	Easy for caregivers to administer
	•	Supports adherence
Optimizes supply chain management	•	Easy to transport
	•	Easy to store
	•	Easy to distribute
Available for resource-limited settings	•	Product is licensed/registered for use in resource-limited settings
	•	Reliable supply of product
Comparative cost	•	Cost should not be a deciding factor; however comparative cost of formulations of the same drug/drug combination should be considered

SRA, stringent regulatory agencies; WHO PQ, WHO pre-qualification programme.

This list is endorsed by major implementers and purchasers of paediatric ARVs such as PEPFAR, UNICEF and the Global Fund, and enables consolidation of demand around optimal products. Procurement is coordinated through the Paediatric ARV procurement working group, a consortium (composed of the Global Fund, PEPFAR, CHAI and UNICEF) which pools orders from the various procurement entities (including some national governments) and schedules production with manufacturers.

### Remaining challenges and new directions

In late December 2014, WHO convened a second meeting on paediatric ARV drug optimization (PADO 2) with the goal of informing the WHO 2015 Guidelines development process, reviewing the list of mid- and long-term priorities for drug and formulations development as well as identifying additional research gaps in this area. Three main challenges were identified. First, as consideration is given to testing HIV-exposed infants at birth and more evidence is gathered on the risk and benefits of very early treatment to limit the viral reservoir [20], there is a need to identify safe and effective ARV options for newborns (less than four weeks, including premature children). A second challenge lies in identifying the most suitable regimen for adolescents who are generally at greater risk of poor adherence and consequent treatment failure and drug resistance development [21]. Finally, there is a need to improve second- and third-line options for children. The management of treatment failure is still very limited in resource-limited settings because of limited drug availability, limited access to viral load, and a general lack of guidance in national treatment guidelines, with consequent delays in switching from failing regimens [22] and accumulation of resistance mutations.

As a guiding principle, PADO 2 participants reiterated the importance of maintaining a public health approach focused on harmonization and simplification of regimens to minimize fragmentation and in turn support sustainability of supply.

To date, while there is robust, randomized, trial data to support the use of LPV/r over NVP in children less than three years, for neonates under two weeks of age NVP remains the preferred choice as there is no approved dose or formulation that can be used in this age group. Unfortunately, there is only sparse PK and safety data for other drugs in newborns (especially low birth weight or premature infants).

For adolescents, consideration was given to providing an integrase-inhibitor-based, first-line regimen to improve adherence, but it was recognized that drug optimization for adolescents needed to focus on maintaining simplification and harmonization with adult regimens. The treatment of adolescents infected horizontally may pose specific challenges, such as further reduction of adherence as a result of lack of support and poor access to health services as a result of stigma and discrimination. While perinatally infected adolescents face issues related to disclosure, treatment fatigue or stigma within their schools, homes and communities, those who acquire HIV through horizontal transmission may have specific challenges for adherence especially if they are infected as a result of sexual abuse or injection drug use.

Integrase inhibitors may also have a role in second-line regimens, particularly for children failing treatment after starting a PI-based regimen. Defining the optimal sequencing of this class of drugs and improving availability are key priorities.

The development of once-a-day FDC regimen continues to be a priority. For first-line regimen, the triple FDC ABC/3TC/EFV remains a critical product for children aged 3–10 years. Raltegravir (RAL) for infants could be of importance due to the time lag expected for full approval of dolutegravir (DTG), a potentially more potent integrase inhibitor with the advantage of a once-daily administration. For second- and third-line regimens, DRV/r or ATV/r co-formulations are key products still missing. DTG-based FDCs are of particular interest for adolescents, for whom DTG is already licensed [23]. Infants and small children (<3 years and 10 kg) currently have very limited ART options particularly in TB co-infection where drug–drug interactions may be difficult to manage. Finally, the improved toxicity profile of tenofovir alafenamide fumarate (TAF) [24] compared to TDF reported in trials among adults could make it an attractive option, given the concerns for bone toxicity and growth associated with the use of TDF in children. An accelerated paediatric development plan is needed for this drug.

### Pushing forward treatment optimization

Since the early 2000s when the only option for treating children was to split adult tablets, tremendous progress has been made to ensure access to better, simpler treatments for children. Yet more needs to be done.

Recent data on the immunological benefits of early therapy, including the elusive prospect of a cure, suggest that children should start treatment as soon as their infection status is confirmed. Innovative strategies have been developed to study the PK of new ARVs in newborn infants taking advantage of the fact that new regimens are being evaluated in pregnant women. Studies of drug clearance among infants who experience transplacental exposure to medications have provided essential data on their PK early in life. Originator companies have been willing to share their intellectual property rights for drugs for children, but there remains a need for companies to pursue the paediatric development of their newer drugs all the way down to infancy as individual drugs (rather than the sole development of FDCs of drugs owned by the same company). Advocacy is needed to address the lack of paediatric investigation plans for single entities that would enable development of generic FDCs and to ensure that those plans are completed as quickly as possible. While PK modelling can fill gaps and allow development of some of the needed FDCs identified at PADO 2, the availability of clinical data to verify weight-band dosing and promote harmonization of weight-bands across multiple products remains essential for the development of new products.

Clinical trials must be performed to determine the optimal sequence of new drugs from birth to adolescence. A good example is the integrase inhibitors. These potent new drugs appear to be very safe, yet they have to prove superiority or at least equivalence to the drugs currently recommended. Demonstrating the relative efficacy, safety and robustness of integrase-inhibitor-based regimens in comparison to protease inhibitors or NNRTI-based regimens will require two to three years. Adaptive trial designs could support the fast-tracking of head-to-head comparisons of new drugs as they become available [25].

Regulators and ethics committees are key actors in efforts to improve access to new, better therapies for children. They need to see their role as not only protecting patients, but also facilitating and expediting paediatric clinical research, without which children may still lag far behind adults. Efforts towards joint approval of drugs at the regional level represent an important step forward.

While adolescents are the first to benefit from the improvements of therapy for adults, there is a need to consider new formulations adapted to their needs. Very short interruptions, such as weekends off drugs [26], provided the drugs have a long half-life, could improve adherence in this group, and long-acting combinations that require bi-weekly or monthly dosing would be ideal during adolescent years.

## Conclusions

The priority for the future is to improve access to ART at the two ends of the age spectrum – infants and adolescents – where the treatment gap is greatest, and optimize drug sequencing with better use of available medicines for second and third line. More efforts are needed to develop FDCs that allow simplified weight-band dosing and minimize the number of products needed, which in turn will improve procurement and availability.

Future efforts will require continuous collaboration and coordination, and the promotion of innovative approaches to accelerate access to new drugs. While significant progress has been made, additional efforts are needed to ensure that treatment targets are reached by 2020. Originator pharmaceutical companies, generic producers and regulators all need to work together to meet with the needs of children in resource-limited settings.
